# Gene expression profiles of specific chicken skeletal muscles

**DOI:** 10.1038/s41597-022-01668-w

**Published:** 2022-09-08

**Authors:** Hua Kui, Bo Ran, Maosen Yang, Xin Shi, Yingyu Luo, Yujie Wang, Tao Wang, Diyan Li, Surong Shuai, Mingzhou Li

**Affiliations:** 1 Institute of Animal Genetics and Breeding, College of Animal Science and Technology, Sichuan Agricultural University, Chengdu, 611130 Sichuan China; 2 School of Pharmacy, Chengdu University, Chengdu, 610106 Sichuan China

**Keywords:** Gene expression, Animal breeding

## Abstract

The chicken provides large amounts of protein for the human diet and is also used as a model organism for biomedical research. Increasing meat production is an important goal in the poultry industry and skeletal muscles have highly diverse origins, shapes, metabolic features, and physical functions. Previous gene expression atlases have largely ignored the differences among diverse types of skeletal muscles; therefore, comprehensive transcriptional maps of all skeletal muscles are needed to improve meat production traits. In this study, we sequenced 58 samples from 10 different skeletal muscles of 42-day-old White Plymouth Rock chickens. We also measured myofiber diameter and generated myofiber-type datasets of these 10 tissues. We generated 418.4 Gb high-quality bulk RNA-Seq data from four or six biological replicates of each skeletal muscle (four replicates from extraocular samples) (approximately 7.4 Gb per sample). This dataset provides valuable information for understanding the muscle fiber characteristics of White Plymouth Rock chickens. Furthermore, our data can be used as a model for heterogeneity analysis between tissues with similar properties.

## Abstract (summary)

**Table:** 

Measurement(s)	Gene expression profiles of specific chicken skeletal muscles • cross section area of chicken skeletal muscles and type I muscle fiber quantity
Technology Type(s)	RNA sequence • Histological Procedure
Factor Type(s)	different skeletal muscles
Sample Characteristic - Organism	Gallus gallus
Sample Characteristic - Environment	farm

## Background & Summary

Skeletal muscles together constitute the largest organ in vertebrate bodies^1^ and in chicken, skeletal muscles constitute nearly 50% of the total body weight^2^. Skeletal muscles have highly diverse origins, shapes, metabolic features, and physical functions^3^, but their gene expression patterns remain largely unexplored^4–9^. However, there have been few systematic analyses of transcriptional diversity in chicken skeletal muscle. Chicken (*Gallus gallus domesticus*) is the most abundant domesticated animal in the world^10^ and broiler chickens are considered a key source of protein in the human diet^11^. In addition, the chicken is used as a model for studying skeletal muscle^12^.

**Fig. 1:**
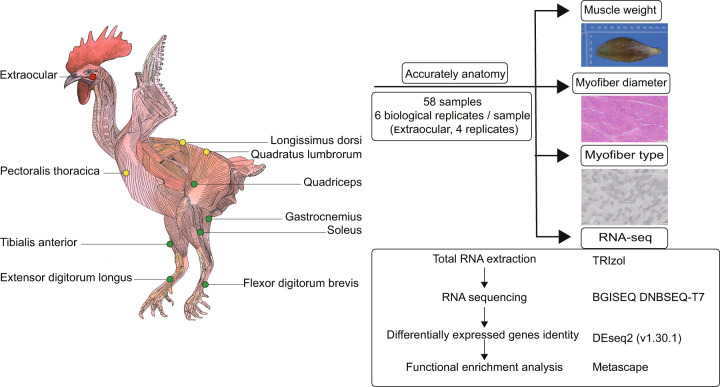
Depiction of chicken skeletal muscle anatomy, sample collection, histological analysis, RNA sequencing, and data analysis.

To characterize transcriptome variability in known muscle-specific physiological activities and to identify key transcripts underlying economically important phenotypes in chickens, notably, meat production, we sequenced 58 paired-end RNA-Seq libraries of 10 skeletal muscle tissues from different anatomical regions of White Plymouth Rock chickens (Fig. 1; Supplementary Table 1). In addition, we performed histological analysis of skeletal muscles, such as determining the ratio of muscle weight/body weight, fiber diameter, myofiber cross section area (CSA), and the ratio of fiber type I to muscle section area.

**Fig. 2:**
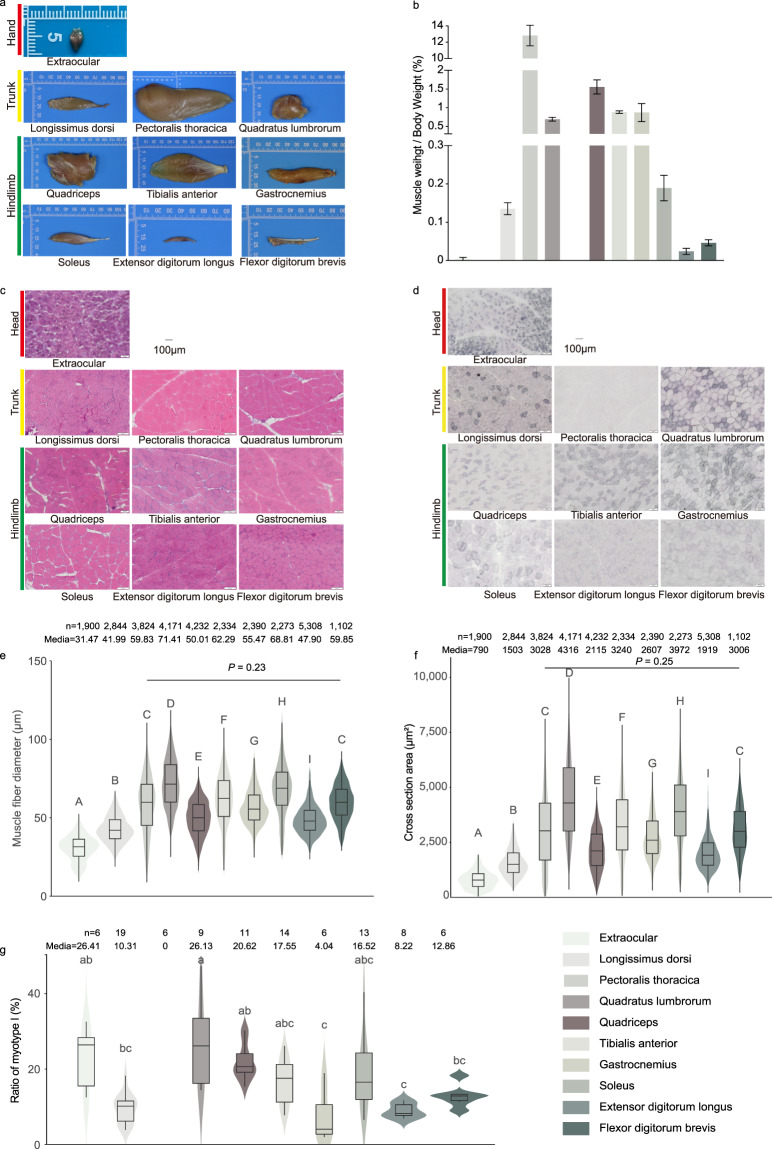
Anatomy and histological analysis of chicken skeletal muscles. (**a**) Representative images of ten chicken skeletal muscles. (**b**) The muscle weight to body weight ratio of individual skeletal muscles. The values are expressed as the mean ± SD. (**c**) Hematoxylin and eosin staining of muscles. (**d**) Succinate dehydrogenase staining of muscles. (**e**) Muscle fiber diameter. (**f**) Cross section area of myofibers. Different uppercase letters indicate highly statistically significant differences (*P* < 0.01), different lowercase letters indicate statistically significant differences (0.01 < *P* < 0.05), and the same letters indicate no statistically significant difference (*P* > 0.05). (**g**) The ratio of myofiber type I area to muscle section area. Different letters indicate a significant difference at *p* < 0.05 by a one-way ANOVA.

To investigate the properties of chicken skeletal muscles, we accurately dissected chicken skeletal muscles (Fig. 1 left panel; Fig. 2a) by referencing anatomical atlas of chicken^13^. Ten skeletal muscles were selected according to the broadest interest in skeletal muscles by a Google Forms survey^14^ and economically important traits. Muscles were classified into three anatomical regions (Fig. 1a, left panel); one muscle from the head (extraocular), three from the trunk (longissimus dorsi, pectoralis thoracica, and quadratus lumbrorum), and six from the leg (quadriceps, tibialis anterior, gastrocnemius, soleus, extensor digitorum longus, and flexor digitorum brevis). We dissected, weighed, photographed, and RNA-sequenced the 10 muscles (Fig. 1, right panel).

The muscle weight/body weight ratio of the pectoralis thoracica muscle (12.82%) was significantly higher compared with the other nine muscles (Fig. 2a,b; Supplementary Tables 2-3). The median diameter (71.41 μm) and CSA (4,136.00 μm^2^) of the quadratus lumbrorum muscle were significantly higher (*P* < 0.01) than those of the other nine muscles (Fig. 2c–f). The fiber type I to muscle section area ratio of the extraocular (26.41%) and quadratus lumbrorum (26.13%) muscles were both higher than those of the other eight muscles (Fig. 2g). There were no type I fibers in the pectoralis thoracica muscle (Fig. 2g).

**Fig. 3:**
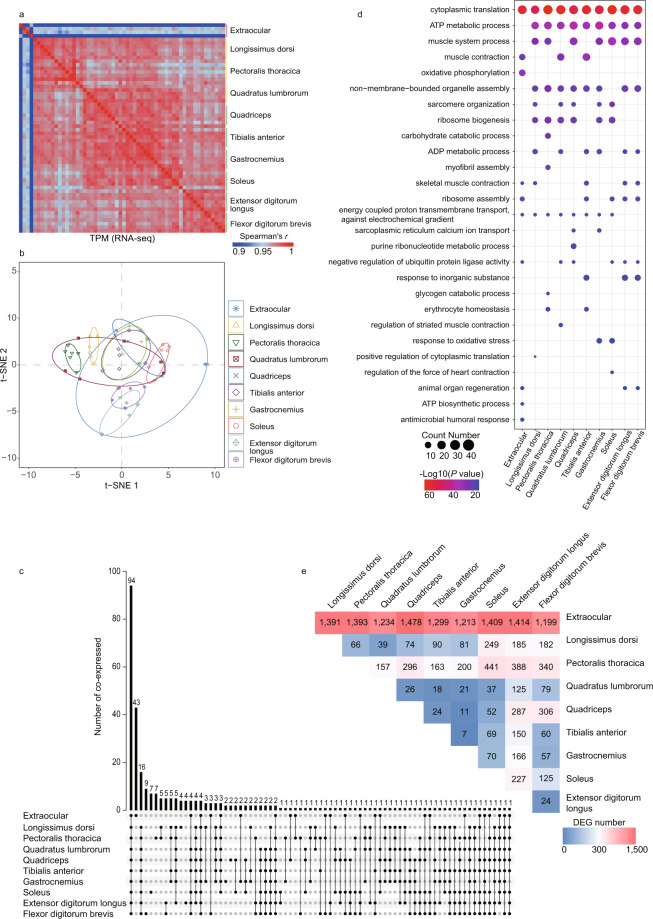
Expression profiles of chicken skeletal muscles. (**a**) Spearman’s *r* heatmap for gene expression profiles of the 58 samples. (**b**) t-Distributed Stochastic Neighbor Embedding analysis (t-SNE) of gene expression profiles for all 58 samples. (**c**) UpSet plot of the top 1% most highly expressed genes (n = 172) in each muscle. (**d**) The top 10 significantly enriched Gene Ontology-Biological Process (GO-BP) terms of the top 1% most highly expressed genes in each muscle. **(e)** Heatmap of numbers of differentially expressed genes (DEGs) in pairwise comparisons among the ten muscles.

Different skeletal muscles showed extensive transcriptional heterogeneity. Only half (94, or 54.65%) of the most abundant genes (the top 1%; 172 genes) for a given skeletal muscle were common in all ten muscles (Fig. 3c). These highly expressed genes (Fig. 3c; Supplementary Table 4) were involved in GO or KEGG, the commonly categories including ‘cytoplasmic translation’, and ‘ATP metabolic’, and the tissue-specific categories such as ‘oxidative phosphorylation’ for the extraocular muscle (Fig. 3d). We performed pairwise differential expression analysis for the 10 muscles and found that the extraocular muscle had the greatest number of differentially expressed genes (DEGs) (Fig. 3e).

## Methods

### Ethics statement

All experimental procedures with chickens were performed according to the Guidelines for Experimental Animals established by the Ministry of Science and Technology (Beijing, China, revised in March 2017). Ethical approval on animal survival was given by the Committee of Sichuan Agricultural University (protocol number 2020102003). The experiments were carried out in accordance with the approved guidelines.

### Animals and sample collection

Healthy male White Plymouth Rock chickens at 42 days of age were obtained from the Sichuan Agriculture University poultry breeding farm (Ya’an, China). Chickens were euthanized via the intravenous injection of 2% pentobarbital sodium (25 mg/kg body weight). To comprehensively survey the chicken transcriptome, 58 samples from 10 skeletal muscles, four or six biological replicates for each skeletal muscle (four replicates for extraocular muscles, six replicates for other nine muscles) were collected. The intact skeletal muscles were carefully dissected and then weighed. The skeletal muscle samples were promptly frozen in liquid nitrogen and stored at −80 °C for subsequent experiments.

### RNA-Seq library construction and sequencing

Total RNA was extracted from each sample using RNAiso Plus reagent (TaKaRa, Otsu, Shiga, Japan) according to the manufacturer’s instructions. The purity of the total RNA was estimated using a NanoDrop 2000 spectrophotometer (Thermo Fisher Scientific, Waltham, MA, USA). The integrity and concentration of total RNA was assessed using the RNA Nano 6000 Assay Kit of the Bioanalyzer 2100 system (Agilent Technologies, CA, USA). Then, the MGIEasy RNA library preparation kit was used to construct 58 poly-A RNA-Seq libraries. These libraries were then sequenced using the BGISEQ DNBSEQ-T7 platform (BGI lnc., Shenzhen, China) with a paired-end sequencing length of 150 bp (PE 150) by Annoroad Gene Technology Co., Ltd (Beijing, China). The SRA accession number for this dataset is PRJNA837345.

### Gene expression analyses

Raw data (raw reads) in fastq format were first processed using WriteFQ software. At the same time, Q20, Q30 and GC content of the clean data were calculated. All the downstream analyses were based on the high-quality clean data (clean data). The clean data were mapped to the chicken reference genome (GRCg6a) using the STAR alignment tool (v.2.7.6a), and gene expression quantified as TPM (transcripts per million) using Kallisto (v.0.44.0) software. Spearman correlations were calculated across the 10 skeletal muscles. DEGs were identified using DEseq 2 (v.1.30.1)^15^ based on the read count data. Significant DEGs were screened with a false discovery rate < 0.05 and |log_2_ fold change| > 1 as cutoffs.

### Functional enrichment analysis

Functional enrichment analyses were performed using Metascape (http://metascape.org)^16^ with default parameters. Chicken genes were converted to human orthologs, and the target gene lists were uploaded as inputs for enrichment. We chose human (*Homo sapiens*) as the target species, and enrichment analysis was performed against all genes in the genome as the background set, with the biological process (BP) of Gene Ontology (GO) as the functional test set. Only GO terms with a *P* value < 0.01 and annotated to ≥2 genes were considered significant.

### Histological analysis

Fresh skeletal muscles were mounted in optimal cutting temperature compound (Sakura) by flash freezing in liquid nitrogen, and 10-μm thick sections cut using a cryosectioning machine (NX-50; Thermo Fisher Scientific). Hematoxylin and eosin (H&E) staining was conducted according to routine protocols^17^. For succinate dehydrogenase (SDH) staining, slides were incubated in SDH staining solution for 35 minutes at 37 °C and then washed in distilled water. Three images per section and six sections from each chicken were analyzed. Micrographs were obtained using a slide scanner (BX61VS; Olympus) and diameter and cross-section area (CSA) were analyzed using Image Pro Plus software (v.6.0). In SDH-stained sections, dark fibers were identified as type I and manually colored red. Conversely, the other myofiber regions were colored green using Photoshop software (v.21.0.0). Then, type I myofiber areas and other fibers were identified using Image J software (v.1.52).

### Statistical analyses

All results in Fig. 2b were expressed as the mean ± SD. The significance of the difference was calculated by a one-way analysis of variance (ANOVA) with Duncan’s post-hoc test in Fig. 2g. The Wilcoxon rank-sum test was used for difference analysis in Fig. 2e,f, *P* < 0.05 was considered significant.

## Data Records

The RNA-Seq data of chicken skeletal muscles have been deposited into the National Center for Biotechnology Information (NCBI) SRA database (Experiments for SRP374834) under BioProject accession number PRJNA837345^18^.

## Technical Validation

### Sequencing quality control

A total of 418.4 Gb of clean sequence was generated from 58 libraries with an average of 7.4 Gb for each sample. Mapping the clean data to the chicken reference genome (GRCg6a) using STAR alignment, showed that the number of raw sequence reads per sample ranged from 23.97 to 18.32 million, with an average of 22.64 million and an average input read length of 300 bp (2 × 150 bp). Uniquely mapped reads averaged 20.31 million, representing 89.69% of the total average reads. A heatmap of correlation showed high reproducibility within biological replicates (Spearman’s *r* > 0.80) (Fig. 3a; Supplementary Table 1). Interestingly, anatomical neighbors generally exhibited more similar patterns of expression than muscles from arbitrarily paired anatomical regions (Fig. 3b).

## Usage Notes

These datasets will provide valuable information for understanding muscle fiber characteristics of White Plymouth Rock chickens. Furthermore, these data can be used as a model for heterogeneity analysis between tissues with similar properties. We only observed the mRNA profiles of White Plymouth Rock chickens, which is a fast-growing breed; however, these data can be used to comprehensively compare fast and slow growing chicken breeds (such as slow growing native chicken breeds) or between broilers and hens.

## Supplementary information

Supplementary Table 1, 2, 3,and 4

## Data Availability

Code files are available from the GitHub repository https://github.com/YMSen/Chicken_skeletal_muscle. All the bioinformatics analyses were performed in R 4.0.3 on x86_64-pc-linux-gnu (64-bit) platform, running under CentOS Linux release 7.9.2009 (Core). The following software packages were used for the analyses: STAR v2.7.0e, kallisto v0.44.0, tximport v1.20.0, tximeta v1.8.5, ggforce v0.3.3, limma v3.48.3, DESeq 2 v1.32.0, pheatmap v1.0.12, ggplot2 v3.3.6, RColorBrewer v1.1–3 and Rtsne v0.15.
