# Supplementary material for: Gene expression profiles of specific chicken skeletal muscles
Source: Sci Data. 2022 Sep 8;9:552. doi: 10.1038/s41597-022-01668-w (PMC9458719; doi:10.1038/s41597-022-01668-w)
Supplement: Supplementary file 1 — Supplementary Table 1, 2, 3,and 4 [file 41597_2022_1668_MOESM1_ESM.pdf]

Supplementary Information

Supplementary Information Table of Contents

Supplementary Table 1 Sequencing data summary of RNA-seq for all 58 samples.

Supplementary Table 2 The weight of chickens.

Supplementary Table 3 The muscle weight to body weight ratio of individual skeletal muscles.

Supplementary Table 4 The top 1% highly expressed genes at each skeletal muscle.

Supplementary Table 1 Sequencing data summary of RNA-seq for all 58 samples

Sample ID	Clean Reads (Mb)	Clean Base (Gb)	Q30(%)
Extraocular_1	48.4	7.2	93.37
Extraocular_4	45.2	6.7	93.12
Extraocular_5	53.4	8	92.81
Extraocular_6	52.6	7.8	93.53
Longissimus dorsi_1	55.7	8.3	92.97
Longissimus dorsi_2	46.8	7	93.02
Longissimus dorsi_3	48.7	7.3	93.31
Longissimus dorsi_4	48.3	7.2	92.85
Longissimus dorsi_5	48.8	7.3	92.87
Longissimus dorsi_6	46.1	6.9	92.52
Pectoralis thoracica_1	49.4	7.4	92.8
Pectoralis thoracica_2	48.4	7.2	92.84
Pectoralis thoracica_3	48.6	7.2	92.83
Pectoralis thoracica_4	48.4	7.2	92.83
Pectoralis thoracica_5	47.4	7.1	93.07
Pectoralis thoracica_6	47.3	7.3	93.5
Quadratus lumbrorum_1	49.6	7.4	93.23
Quadratus lumbrorum_2	48	7.2	92.65
Quadratus lumbrorum_3	52.3	7.8	92.45
Quadratus lumbrorum_4	49	7.3	93.18
Quadratus lumbrorum_5	48.3	7.2	92.77
Quadratus lumbrorum_6	49.8	7.4	92.84
Quadriceps_1	49.5	7.4	92.81
Quadriceps_2	44.7	6.7	92.34
Quadriceps_3	50.5	7.5	92.78
Quadriceps_4	47.3	7.1	92.92
Quadriceps_5	48.2	7.2	92.95
Quadriceps_6	50.9	7.6	92.85
Tibialis anterior_1	46.4	6.9	93.21
Tibialis anterior_2	46	6.9	92.99
Tibialis anterior_3	50.6	7.5	92.78
Tibialis anterior_4	50	7.5	93.13
Tibialis anterior_5	51.3	7.6	92.55
Tibialis anterior_6	47.3	7.1	92.74
Gastrocnemius_1	47.2	7	92.32
Gastrocnemius_2	49.1	7.3	92.38
Gastrocnemius_3	49.3	7.4	92.32
Gastrocnemius_4	50.1	7.5	92.86
Gastrocnemius_5	52.6	7.8	92.94

Continued on next page

Supplementary Table 1 (*continued*) Sequencing data summary of RNA-seq for all 58 samples

Sample ID	Clean Reads (Mb)	Clean Base (Gb)	Q30(%)
Gastrocnemius_6	51.5	7.7	92.87
Soleus_1	49	7.3	93.5
Soleus_2	49.2	7.3	91.53
Soleus_3	48.6	7.2	92.85
Soleus_4	52.3	7.8	93.17
Soleus_5	49.3	7.3	93.04
Soleus_6	50.1	7.5	93.09
Extensor digitorum longus _1	47.5	7.1	92.99
Extensor digitorum longus _2	43	6.4	92.39
Extensor digitorum longus _3	51.3	7.7	93.41
Extensor digitorum longus _4	53.1	7.9	91.94
Extensor digitorum longus _5	49.2	7.3	93.17
Extensor digitorum longus _6	49.7	7.4	92.76
Flexor digitorum brevis _1	52.8	7.9	93.05
Flexor digitorum brevis _2	51.3	7.7	93.09
Flexor digitorum brevis _3	46.6	6.9	92.96
Flexor digitorum brevis _4	49.9	7.4	93.01
Flexor digitorum brevis _5	49.1	7.3	92.87
Flexor digitorum brevis _6	48.4	7.2	93.18

Supplementary Table 2 The weight of chickens

Sample name	Body weight (kg)
Chicken_1	2785.00
Chicken_2	2590.00
Chicken_3	2700.00
Chicken_4	2860.00
Chicken_5	3085.00
Chicken_6	3060.00

Supplementary Table 3 The muscle weight to body weight ratio of individual skeletal muscles

	Muscle weight (g)						Muscle weight/Body weight (%)	Standard Deviation
	Chicken_1	Chicken_2	Chicken_3	Chicken_4	Chicken_5	Chicken_6		
**Extraocular**	0.04	0.33	0.04	0.04	0.05	0.04	0.003317332	0.004583413
**Longissimus dorsi**	4.46	3.26	3.24	4.20	4.22	3.76	0.13542204	0.01568575
**Pectoralis thoracica**	410.00	350.00	310.00	346.62	361.10	410.00	12.82333601	1.261564277
**Quadratus lumbrorum**	18.54	18.36	16.56	19.80	21.86	23.42	0.69236332	0.050637417
**Quadriceps**	53.04	39.38	42.94	38.52	44.70	46.70	1.556210912	0.189727318
**Tibialis anterior**	26.28	22.34	23.06	25.72	26.74	26.58	0.882491962	0.033658752
**Gastrocnemius**	10.64	25.94	24.36	28.12	29.18	30.66	0.869476328	0.241837344
**Soleus**	5.54	5.42	5.52	3.54	6.52	5.78	0.189440664	0.0331663
**Extensor digitorum longus**	0.38	0.50	0.84	0.62	1.10	0.64	0.023718402	0.008125882

Supplementary Table 4 The top 1% highly expressed genes at each skeletal muscle

Extraocular	Longissimus dorsi	Pectoralis thoracica	Quadratus lumbrorum	Quadriceps	Tibialis anterior	Gastrocnemius	Soleus	Extensor digitorum longus	Flexor digitorum brevis
ENSGALG000 00000313	ENSGALG000 00000474	ENSGALG000 00000295	ENSGALG000 00000313	ENSGALG000 00000313	ENSGALG000 00000313	ENSGALG000 00000313	ENSGALG000 00000474	ENSGALG000 00000313	ENSGALG000 00000313
ENSGALG000 00000474	ENSGALG000 00000812	ENSGALG000 00000474	ENSGALG000 00000474	ENSGALG000 00000474	ENSGALG000 00000474	ENSGALG000 00000474	ENSGALG000 00000812	ENSGALG000 00000474	ENSGALG000 00000474
ENSGALG000 00000812	ENSGALG000 00001096	ENSGALG000 00000812	ENSGALG000 00000812	ENSGALG000 00000812	ENSGALG000 00000812	ENSGALG000 00000812	ENSGALG000 00001096	ENSGALG000 00000812	ENSGALG000 00000812
ENSGALG000 00001096	ENSGALG000 00001446	ENSGALG000 00001096	ENSGALG000 00001096	ENSGALG000 00001096	ENSGALG000 00001096	ENSGALG000 00001096	ENSGALG000 00001330	ENSGALG000 00001446	ENSGALG000 00001096
ENSGALG000 00001330	ENSGALG000 00001465	ENSGALG000 00001446	ENSGALG000 00001330	ENSGALG000 00001330	ENSGALG000 00001446	ENSGALG000 00001330	ENSGALG000 00001465	ENSGALG000 00001459	ENSGALG000 00001446
ENSGALG000 00001446	ENSGALG000 00001564	ENSGALG000 00001459	ENSGALG000 00001446	ENSGALG000 00001446	ENSGALG000 00001465	ENSGALG000 00001446	ENSGALG000 00001564	ENSGALG000 00001465	ENSGALG000 00001459
ENSGALG000 00001465	ENSGALG000 00001634	ENSGALG000 00001465	ENSGALG000 00001459	ENSGALG000 00001459	ENSGALG000 00001634	ENSGALG000 00001459	ENSGALG000 00001634	ENSGALG000 00001634	ENSGALG000 00001465
ENSGALG000 00001634	ENSGALG000 00001793	ENSGALG000 00001634	ENSGALG000 00001465	ENSGALG000 00001465	ENSGALG000 00001658	ENSGALG000 00001465	ENSGALG000 00001658	ENSGALG000 00001658	ENSGALG000 00001634
ENSGALG000 00001658	ENSGALG000 00001926	ENSGALG000 00001658	ENSGALG000 00001634	ENSGALG000 00001634	ENSGALG000 00001709	ENSGALG000 00001634	ENSGALG000 00001926	ENSGALG000 00001709	ENSGALG000 00001658
ENSGALG000 00001793	ENSGALG000 00001977	ENSGALG000 00001709	ENSGALG000 00001658	ENSGALG000 00001658	ENSGALG000 00001793	ENSGALG000 00001658	ENSGALG000 00001977	ENSGALG000 00001793	ENSGALG000 00001709
ENSGALG000 00002157	ENSGALG000 00001992	ENSGALG000 00001926	ENSGALG000 00001709	ENSGALG000 00001709	ENSGALG000 00001926	ENSGALG000 00001709	ENSGALG000 00001992	ENSGALG000 00001926	ENSGALG000 00001793
ENSGALG000 00002160	ENSGALG000 00002160	ENSGALG000 00001977	ENSGALG000 00001793	ENSGALG000 00001793	ENSGALG000 00001977	ENSGALG000 00001793	ENSGALG000 00002160	ENSGALG000 00001977	ENSGALG000 00001926

Continued on next page

Supplementary Table 4 (*continued*) The top 1% highly expressed genes at each skeletal muscle

Extraocular	Longissimus dorsi	Pectoralis thoracica	Quadratus lumbrorum	Quadriceps	Tibialis anterior	Gastrocnemius	Soleus	Extensor digitorum longus	Flexor digitorum brevis
ENSGALG00000002813	ENSGALG00000002813	ENSGALG00000001992	ENSGALG00000001926	ENSGALG00000001926	ENSGALG00000001992	ENSGALG00000001926	ENSGALG00000002495	ENSGALG00000001992	ENSGALG00000001992
ENSGALG00000002837	ENSGALG00000002868	ENSGALG00000002157	ENSGALG00000001977	ENSGALG00000001977	ENSGALG00000002157	ENSGALG00000001977	ENSGALG00000002813	ENSGALG00000002157	ENSGALG00000002157
ENSGALG00000002868	ENSGALG00000002907	ENSGALG00000002160	ENSGALG00000001992	ENSGALG00000001992	ENSGALG00000002160	ENSGALG00000001992	ENSGALG00000002837	ENSGALG00000002160	ENSGALG00000002160
ENSGALG00000002907	ENSGALG00000003197	ENSGALG00000002813	ENSGALG00000002157	ENSGALG00000002160	ENSGALG00000002813	ENSGALG00000002157	ENSGALG00000002868	ENSGALG00000002813	ENSGALG00000002771
ENSGALG00000003197	ENSGALG00000003226	ENSGALG00000002868	ENSGALG00000002160	ENSGALG00000002813	ENSGALG00000002837	ENSGALG00000002160	ENSGALG00000002907	ENSGALG00000002837	ENSGALG00000002813
ENSGALG00000003521	ENSGALG00000003521	ENSGALG00000002907	ENSGALG00000002813	ENSGALG00000002837	ENSGALG00000002868	ENSGALG00000002813	ENSGALG00000003197	ENSGALG00000002868	ENSGALG00000002837
ENSGALG00000003966	ENSGALG00000003778	ENSGALG00000003197	ENSGALG00000002837	ENSGALG00000002868	ENSGALG00000002907	ENSGALG00000002837	ENSGALG00000003226	ENSGALG00000002907	ENSGALG00000002868
ENSGALG00000004107	ENSGALG00000003966	ENSGALG00000003521	ENSGALG00000002868	ENSGALG00000002907	ENSGALG00000003197	ENSGALG00000002868	ENSGALG00000003521	ENSGALG00000003015	ENSGALG00000002907
ENSGALG00000004184	ENSGALG00000004509	ENSGALG00000003966	ENSGALG00000002907	ENSGALG00000003197	ENSGALG00000003226	ENSGALG00000002907	ENSGALG00000003966	ENSGALG00000003197	ENSGALG00000003015
ENSGALG00000004509	ENSGALG00000004521	ENSGALG00000004509	ENSGALG00000003197	ENSGALG00000003226	ENSGALG00000003521	ENSGALG00000003197	ENSGALG00000004509	ENSGALG00000003226	ENSGALG00000003197
ENSGALG00000004521	ENSGALG00000004818	ENSGALG00000004521	ENSGALG00000003521	ENSGALG00000003521	ENSGALG00000003966	ENSGALG00000003521	ENSGALG00000004521	ENSGALG00000003521	ENSGALG00000003226
ENSGALG00000004582	ENSGALG00000004848	ENSGALG00000004818	ENSGALG00000003966	ENSGALG00000003966	ENSGALG00000004184	ENSGALG00000003966	ENSGALG00000004818	ENSGALG00000003966	ENSGALG00000003283
ENSGALG00000004594	ENSGALG00000004871	ENSGALG00000004848	ENSGALG00000004184	ENSGALG00000004184	ENSGALG00000004509	ENSGALG00000004044	ENSGALG00000004848	ENSGALG00000004044	ENSGALG00000003521

Continued on next page

Supplementary Table 4 (*continued*) The top 1% highly expressed genes at each skeletal muscle

Extraocular	Longissimus dorsi	Pectoralis thoracica	Quadratus lumbrorum	Quadriceps	Tibialis anterior	Gastrocnemius	Soleus	Extensor digitorum longus	Flexor digitorum brevis
ENSGALG00000004818	ENSGALG00000004952	ENSGALG00000004871	ENSGALG00000004509	ENSGALG00000004509	ENSGALG00000004521	ENSGALG00000004184	ENSGALG00000004871	ENSGALG00000004184	ENSGALG00000003966
ENSGALG00000004871	ENSGALG00000004956	ENSGALG00000004952	ENSGALG00000004521	ENSGALG00000004521	ENSGALG00000004818	ENSGALG00000004509	ENSGALG00000004952	ENSGALG00000004509	ENSGALG00000004107
ENSGALG00000004952	ENSGALG00000005002	ENSGALG00000004956	ENSGALG00000004818	ENSGALG00000004818	ENSGALG00000004848	ENSGALG00000004521	ENSGALG00000004956	ENSGALG00000004521	ENSGALG00000004184
ENSGALG00000005349	ENSGALG00000005226	ENSGALG00000005002	ENSGALG00000004848	ENSGALG00000004848	ENSGALG00000004871	ENSGALG00000004594	ENSGALG00000005002	ENSGALG00000004594	ENSGALG00000004509
ENSGALG00000005490	ENSGALG00000005407	ENSGALG00000005226	ENSGALG00000004871	ENSGALG00000004871	ENSGALG00000004952	ENSGALG00000004818	ENSGALG00000005226	ENSGALG00000004818	ENSGALG00000004521
ENSGALG00000005749	ENSGALG00000005458	ENSGALG00000005407	ENSGALG00000004952	ENSGALG00000004952	ENSGALG00000004956	ENSGALG00000004848	ENSGALG00000005490	ENSGALG00000004848	ENSGALG00000004594
ENSGALG00000005843	ENSGALG00000005490	ENSGALG00000005458	ENSGALG00000005002	ENSGALG00000005002	ENSGALG00000005002	ENSGALG00000004871	ENSGALG00000005749	ENSGALG00000004871	ENSGALG00000004818
ENSGALG00000005922	ENSGALG00000005587	ENSGALG00000005490	ENSGALG00000005226	ENSGALG00000005226	ENSGALG00000005226	ENSGALG00000004952	ENSGALG00000005843	ENSGALG00000004952	ENSGALG00000004848
ENSGALG00000005948	ENSGALG00000005749	ENSGALG00000005587	ENSGALG00000005448	ENSGALG00000005448	ENSGALG00000005448	ENSGALG00000005002	ENSGALG00000005849	ENSGALG00000005226	ENSGALG00000004871
ENSGALG00000006179	ENSGALG00000005843	ENSGALG00000005749	ENSGALG00000005490	ENSGALG00000005490	ENSGALG00000005490	ENSGALG00000005226	ENSGALG00000005922	ENSGALG00000005448	ENSGALG00000004952
ENSGALG00000006572	ENSGALG00000005922	ENSGALG00000005843	ENSGALG00000005749	ENSGALG00000005749	ENSGALG00000005749	ENSGALG00000005448	ENSGALG00000005948	ENSGALG00000005490	ENSGALG00000005226
ENSGALG00000006591	ENSGALG00000005948	ENSGALG00000005922	ENSGALG00000005843	ENSGALG00000005843	ENSGALG00000005843	ENSGALG00000005490	ENSGALG00000006069	ENSGALG00000005749	ENSGALG00000005448
ENSGALG00000006835	ENSGALG00000006069	ENSGALG00000005948	ENSGALG00000005849	ENSGALG00000005849	ENSGALG00000005849	ENSGALG00000005749	ENSGALG00000006179	ENSGALG00000005843	ENSGALG00000005490

Continued on next page

Supplementary Table 4 (*continued*) The top 1% highly expressed genes at each skeletal muscle

**Extraocular**	**Longissimus dorsi**	**Pectoralis thoracica**	**Quadratus lumbrorum**	**Quadriceps**	**Tibialis anterior**	**Gastrocnemius**	**Soleus**	**Extensor digitorum longus**	**Flexor digitorum brevis**
ENSGALG00000007114	ENSGALG00000006179	ENSGALG00000006179	ENSGALG00000005922	ENSGALG00000005922	ENSGALG00000005922	ENSGALG00000005843	ENSGALG00000006190	ENSGALG00000005849	ENSGALG00000005749
ENSGALG00000007205	ENSGALG00000006190	ENSGALG00000006300	ENSGALG00000005948	ENSGALG00000005948	ENSGALG00000005948	ENSGALG00000005849	ENSGALG00000006300	ENSGALG00000005922	ENSGALG00000005843
ENSGALG00000007220	ENSGALG00000006300	ENSGALG00000006572	ENSGALG00000006179	ENSGALG00000006179	ENSGALG00000006069	ENSGALG00000005922	ENSGALG00000006572	ENSGALG00000005948	ENSGALG00000005849
ENSGALG00000007699	ENSGALG00000006572	ENSGALG00000006591	ENSGALG00000006190	ENSGALG00000006190	ENSGALG00000006179	ENSGALG00000005948	ENSGALG00000006591	ENSGALG00000006179	ENSGALG00000005922
ENSGALG00000007863	ENSGALG00000006591	ENSGALG00000006835	ENSGALG00000006300	ENSGALG00000006300	ENSGALG00000006190	ENSGALG00000006179	ENSGALG00000006835	ENSGALG00000006190	ENSGALG00000005948
ENSGALG00000008620	ENSGALG00000006835	ENSGALG00000007114	ENSGALG00000006572	ENSGALG00000006572	ENSGALG00000006300	ENSGALG00000006190	ENSGALG00000007114	ENSGALG00000006300	ENSGALG00000006179
ENSGALG00000009844	ENSGALG00000007114	ENSGALG00000007220	ENSGALG00000006591	ENSGALG00000006591	ENSGALG00000006572	ENSGALG00000006300	ENSGALG00000007220	ENSGALG00000006572	ENSGALG00000006190
ENSGALG00000009963	ENSGALG00000007220	ENSGALG00000007606	ENSGALG00000006835	ENSGALG00000006753	ENSGALG00000006591	ENSGALG00000006572	ENSGALG00000007606	ENSGALG00000006591	ENSGALG00000006300
ENSGALG00000010023	ENSGALG00000007606	ENSGALG00000007699	ENSGALG00000007114	ENSGALG00000006835	ENSGALG00000006835	ENSGALG00000006591	ENSGALG00000007699	ENSGALG00000006835	ENSGALG00000006572
ENSGALG00000010077	ENSGALG00000007699	ENSGALG00000007863	ENSGALG00000007220	ENSGALG00000007114	ENSGALG00000007114	ENSGALG00000006835	ENSGALG00000007863	ENSGALG00000007114	ENSGALG00000006591
ENSGALG00000010124	ENSGALG00000007863	ENSGALG00000007936	ENSGALG00000007606	ENSGALG00000007205	ENSGALG00000007220	ENSGALG00000007114	ENSGALG00000007936	ENSGALG00000007220	ENSGALG00000006835
ENSGALG00000010614	ENSGALG00000007936	ENSGALG00000008066	ENSGALG00000007699	ENSGALG00000007220	ENSGALG00000007606	ENSGALG00000007205	ENSGALG00000008066	ENSGALG00000007383	ENSGALG00000007114
ENSGALG00000011086	ENSGALG00000008066	ENSGALG00000008620	ENSGALG00000007863	ENSGALG00000007606	ENSGALG00000007699	ENSGALG00000007220	ENSGALG00000008620	ENSGALG00000007606	ENSGALG00000007205

Continued on next page

Supplementary Table 4 (*continued*) The top 1% highly expressed genes at each skeletal muscle

Extraocular	Longissimus dorsi	Pectoralis thoracica	Quadratus lumbrorum	Quadriceps	Tibialis anterior	Gastrocnemius	Soleus	Extensor digitorum longus	Flexor digitorum brevis
ENSGALG00000011475	ENSGALG00000008620	ENSGALG00000008677	ENSGALG00000007936	ENSGALG00000007699	ENSGALG00000007863	ENSGALG00000007539	ENSGALG00000008684	ENSGALG00000007699	ENSGALG00000007220
ENSGALG00000011483	ENSGALG00000008684	ENSGALG00000008684	ENSGALG00000008066	ENSGALG00000007863	ENSGALG00000007936	ENSGALG00000007699	ENSGALG00000009076	ENSGALG00000007863	ENSGALG00000007611
ENSGALG00000011551	ENSGALG00000009654	ENSGALG00000009844	ENSGALG00000008620	ENSGALG00000007936	ENSGALG00000008066	ENSGALG00000007863	ENSGALG00000010023	ENSGALG00000008620	ENSGALG00000007699
ENSGALG00000011740	ENSGALG00000010023	ENSGALG00000010023	ENSGALG00000008684	ENSGALG00000008066	ENSGALG00000008620	ENSGALG00000008066	ENSGALG00000010027	ENSGALG00000008684	ENSGALG00000007863
ENSGALG00000012229	ENSGALG00000010027	ENSGALG00000010027	ENSGALG00000010023	ENSGALG00000008620	ENSGALG00000008684	ENSGALG00000008620	ENSGALG00000010077	ENSGALG00000008945	ENSGALG00000008066
ENSGALG00000012522	ENSGALG00000010077	ENSGALG00000010077	ENSGALG00000010027	ENSGALG00000008684	ENSGALG00000010023	ENSGALG00000008684	ENSGALG00000010124	ENSGALG00000009621	ENSGALG00000008620
ENSGALG00000012783	ENSGALG00000010124	ENSGALG00000010124	ENSGALG00000010077	ENSGALG00000009076	ENSGALG00000010027	ENSGALG00000008805	ENSGALG00000010614	ENSGALG00000009641	ENSGALG00000008677
ENSGALG00000013537	ENSGALG00000010614	ENSGALG00000010614	ENSGALG00000010124	ENSGALG00000010023	ENSGALG00000010077	ENSGALG00000009076	ENSGALG00000011016	ENSGALG00000010023	ENSGALG00000008684
ENSGALG00000013990	ENSGALG00000011016	ENSGALG00000011016	ENSGALG00000010614	ENSGALG00000010027	ENSGALG00000010124	ENSGALG00000010023	ENSGALG00000011086	ENSGALG00000010027	ENSGALG00000008806
ENSGALG00000014432	ENSGALG00000011086	ENSGALG00000011086	ENSGALG00000011016	ENSGALG00000010077	ENSGALG00000010614	ENSGALG00000010027	ENSGALG00000011306	ENSGALG00000010077	ENSGALG00000009621
ENSGALG00000014442	ENSGALG00000011306	ENSGALG00000011290	ENSGALG00000011086	ENSGALG00000010124	ENSGALG00000011016	ENSGALG00000010077	ENSGALG00000011475	ENSGALG00000010124	ENSGALG00000009641
ENSGALG00000014833	ENSGALG00000011475	ENSGALG00000011306	ENSGALG00000011290	ENSGALG00000010614	ENSGALG00000011086	ENSGALG00000010124	ENSGALG00000011483	ENSGALG00000010614	ENSGALG00000010023
ENSGALG00000015082	ENSGALG00000011483	ENSGALG00000011475	ENSGALG00000011306	ENSGALG00000011086	ENSGALG00000011306	ENSGALG00000010614	ENSGALG00000011715	ENSGALG00000011016	ENSGALG00000010027

Continued on next page

Supplementary Table 4 (*continued*) The top 1% highly expressed genes at each skeletal muscle

Extraocular	Longissimus dorsi	Pectoralis thoracica	Quadratus lumbrorum	Quadriceps	Tibialis anterior	Gastrocnemius	Soleus	Extensor digitorum longus	Flexor digitorum brevis
ENSGALG00000015195	ENSGALG00000011715	ENSGALG00000011483	ENSGALG00000011475	ENSGALG00000011306	ENSGALG00000011475	ENSGALG00000011086	ENSGALG00000012211	ENSGALG00000011086	ENSGALG00000010077
ENSGALG00000015339	ENSGALG00000012211	ENSGALG00000012211	ENSGALG00000011483	ENSGALG00000011475	ENSGALG00000011483	ENSGALG00000011290	ENSGALG00000012229	ENSGALG00000011274	ENSGALG00000010124
ENSGALG00000015372	ENSGALG00000012229	ENSGALG00000012229	ENSGALG00000011715	ENSGALG00000011483	ENSGALG00000011740	ENSGALG00000011306	ENSGALG00000012522	ENSGALG00000011306	ENSGALG00000010614
ENSGALG00000015602	ENSGALG00000012612	ENSGALG00000012612	ENSGALG00000011740	ENSGALG00000011715	ENSGALG00000012211	ENSGALG00000011475	ENSGALG00000012541	ENSGALG00000011475	ENSGALG00000011086
ENSGALG00000015617	ENSGALG00000012783	ENSGALG00000013069	ENSGALG00000012211	ENSGALG00000011740	ENSGALG00000012229	ENSGALG00000011483	ENSGALG00000012612	ENSGALG00000011715	ENSGALG00000011274
ENSGALG00000015917	ENSGALG00000013414	ENSGALG00000013414	ENSGALG00000012229	ENSGALG00000011991	ENSGALG00000012541	ENSGALG00000011715	ENSGALG00000012783	ENSGALG00000011740	ENSGALG00000011290
ENSGALG00000016127	ENSGALG00000013990	ENSGALG00000013990	ENSGALG00000012541	ENSGALG00000012211	ENSGALG00000012612	ENSGALG00000011740	ENSGALG00000013414	ENSGALG00000011991	ENSGALG00000011306
ENSGALG00000017299	ENSGALG00000014432	ENSGALG00000014432	ENSGALG00000012612	ENSGALG00000012229	ENSGALG00000012783	ENSGALG00000011991	ENSGALG00000013990	ENSGALG00000012211	ENSGALG00000011475
ENSGALG00000025842	ENSGALG00000014442	ENSGALG00000014442	ENSGALG00000012783	ENSGALG00000012541	ENSGALG00000013414	ENSGALG00000012211	ENSGALG00000014432	ENSGALG00000012229	ENSGALG00000011740
ENSGALG00000026300	ENSGALG00000014463	ENSGALG00000014463	ENSGALG00000013414	ENSGALG00000012783	ENSGALG00000013537	ENSGALG00000012229	ENSGALG00000014442	ENSGALG00000012541	ENSGALG00000011991
ENSGALG00000026383	ENSGALG00000014526	ENSGALG00000014526	ENSGALG00000013537	ENSGALG00000013414	ENSGALG00000013990	ENSGALG00000012541	ENSGALG00000014463	ENSGALG00000012783	ENSGALG00000012229
ENSGALG00000026490	ENSGALG00000014848	ENSGALG00000014848	ENSGALG00000013990	ENSGALG00000013537	ENSGALG00000014432	ENSGALG00000012712	ENSGALG00000014526	ENSGALG00000013414	ENSGALG00000012541
ENSGALG00000026970	ENSGALG00000015018	ENSGALG00000015018	ENSGALG00000014432	ENSGALG00000013990	ENSGALG00000014442	ENSGALG00000012783	ENSGALG00000014848	ENSGALG00000013537	ENSGALG00000012783

Continued on next page

Supplementary Table 4 (*continued*) The top 1% highly expressed genes at each skeletal muscle

Extraocular	Longissimus dorsi	Pectoralis thoracica	Quadratus lumbrorum	Quadriceps	Tibialis anterior	Gastrocnemius	Soleus	Extensor digitorum longus	Flexor digitorum brevis
ENSGALG00000026978	ENSGALG00000015082	ENSGALG00000015082	ENSGALG00000014442	ENSGALG00000014432	ENSGALG00000014463	ENSGALG00000013414	ENSGALG00000015018	ENSGALG00000013990	ENSGALG00000013414
ENSGALG00000027035	ENSGALG00000015339	ENSGALG00000015195	ENSGALG00000014463	ENSGALG00000014442	ENSGALG00000014526	ENSGALG00000013537	ENSGALG00000015082	ENSGALG00000014432	ENSGALG00000013537
ENSGALG00000027142	ENSGALG00000015372	ENSGALG00000015339	ENSGALG00000014526	ENSGALG00000014463	ENSGALG00000014848	ENSGALG00000013990	ENSGALG00000015339	ENSGALG00000014442	ENSGALG00000013990
ENSGALG00000027323	ENSGALG00000015602	ENSGALG00000015372	ENSGALG00000014833	ENSGALG00000014526	ENSGALG00000015018	ENSGALG00000014432	ENSGALG00000015372	ENSGALG00000014463	ENSGALG00000014432
ENSGALG00000027583	ENSGALG00000015617	ENSGALG00000015602	ENSGALG00000015018	ENSGALG00000014833	ENSGALG00000015082	ENSGALG00000014442	ENSGALG00000015602	ENSGALG00000014526	ENSGALG00000014442
ENSGALG00000027963	ENSGALG00000015917	ENSGALG00000015617	ENSGALG00000015082	ENSGALG00000014848	ENSGALG00000015195	ENSGALG00000014463	ENSGALG00000015617	ENSGALG00000014833	ENSGALG00000014463
ENSGALG00000028520	ENSGALG00000016127	ENSGALG00000015917	ENSGALG00000015195	ENSGALG00000015018	ENSGALG00000015339	ENSGALG00000014526	ENSGALG00000015917	ENSGALG00000015018	ENSGALG00000014833
ENSGALG00000028537	ENSGALG00000016342	ENSGALG00000016127	ENSGALG00000015339	ENSGALG00000015082	ENSGALG00000015372	ENSGALG00000014833	ENSGALG00000016127	ENSGALG00000015082	ENSGALG00000015018
ENSGALG00000028872	ENSGALG00000016954	ENSGALG00000016232	ENSGALG00000015372	ENSGALG00000015195	ENSGALG00000015602	ENSGALG00000015018	ENSGALG00000016342	ENSGALG00000015195	ENSGALG00000015082
ENSGALG00000029500	ENSGALG00000017168	ENSGALG00000016342	ENSGALG00000015602	ENSGALG00000015339	ENSGALG00000015617	ENSGALG00000015082	ENSGALG00000017168	ENSGALG00000015339	ENSGALG00000015195
ENSGALG00000029660	ENSGALG00000017299	ENSGALG00000016954	ENSGALG00000015617	ENSGALG00000015372	ENSGALG00000015917	ENSGALG00000015195	ENSGALG00000017299	ENSGALG00000015372	ENSGALG00000015339
ENSGALG00000029837	ENSGALG00000023819	ENSGALG00000017168	ENSGALG00000015917	ENSGALG00000015602	ENSGALG00000016127	ENSGALG00000015339	ENSGALG00000017380	ENSGALG00000015617	ENSGALG00000015372
ENSGALG00000030436	ENSGALG00000023834	ENSGALG00000017299	ENSGALG00000016127	ENSGALG00000015617	ENSGALG00000016232	ENSGALG00000015372	ENSGALG00000019157	ENSGALG00000015917	ENSGALG00000015617

Continued on next page

Supplementary Table 4 (*continued*) The top 1% highly expressed genes at each skeletal muscle

Extraocular	Longissimus dorsi	Pectoralis thoracica	Quadratus lumbrorum	Quadriceps	Tibialis anterior	Gastrocnemius	Soleus	Extensor digitorum longus	Flexor digitorum brevis
ENSGALG00000030878	ENSGALG00000025842	ENSGALG00000023294	ENSGALG00000016954	ENSGALG00000015917	ENSGALG00000016342	ENSGALG00000015602	ENSGALG00000025842	ENSGALG00000016127	ENSGALG00000015917
ENSGALG00000030886	ENSGALG00000026077	ENSGALG00000023819	ENSGALG00000017168	ENSGALG00000016127	ENSGALG00000017168	ENSGALG00000015617	ENSGALG00000026077	ENSGALG00000016826	ENSGALG00000016127
ENSGALG00000031496	ENSGALG00000026300	ENSGALG00000026077	ENSGALG00000017299	ENSGALG00000017168	ENSGALG00000017299	ENSGALG00000015917	ENSGALG00000026300	ENSGALG00000016954	ENSGALG00000016232
ENSGALG00000032002	ENSGALG00000026449	ENSGALG00000026300	ENSGALG00000023819	ENSGALG00000017299	ENSGALG00000019157	ENSGALG00000016127	ENSGALG00000026383	ENSGALG00000017168	ENSGALG00000016775
ENSGALG00000032079	ENSGALG00000026490	ENSGALG00000026383	ENSGALG00000025842	ENSGALG00000017380	ENSGALG00000023819	ENSGALG00000016351	ENSGALG00000026490	ENSGALG00000017299	ENSGALG00000017168
ENSGALG00000032142	ENSGALG00000026553	ENSGALG00000026449	ENSGALG00000026300	ENSGALG00000019157	ENSGALG00000025842	ENSGALG00000017168	ENSGALG00000026553	ENSGALG00000019157	ENSGALG00000017299
ENSGALG00000032456	ENSGALG00000027035	ENSGALG00000026490	ENSGALG00000026383	ENSGALG00000025842	ENSGALG00000026300	ENSGALG00000017299	ENSGALG00000027035	ENSGALG00000023819	ENSGALG00000017330
ENSGALG00000032465	ENSGALG00000027583	ENSGALG00000026978	ENSGALG00000026490	ENSGALG00000025999	ENSGALG00000026383	ENSGALG00000017380	ENSGALG00000027323	ENSGALG00000025842	ENSGALG00000019157
ENSGALG00000032595	ENSGALG00000027771	ENSGALG00000027035	ENSGALG00000026978	ENSGALG00000026300	ENSGALG00000026490	ENSGALG00000019157	ENSGALG00000027583	ENSGALG00000026300	ENSGALG00000023819
ENSGALG00000033446	ENSGALG00000027963	ENSGALG00000027142	ENSGALG00000027035	ENSGALG00000026383	ENSGALG00000027035	ENSGALG00000023772	ENSGALG00000027771	ENSGALG00000026383	ENSGALG00000025842
ENSGALG00000034083	ENSGALG00000028537	ENSGALG00000027771	ENSGALG00000027583	ENSGALG00000026490	ENSGALG00000027583	ENSGALG00000023819	ENSGALG00000027963	ENSGALG00000026490	ENSGALG00000026300
ENSGALG00000035079	ENSGALG00000028612	ENSGALG00000027872	ENSGALG00000027771	ENSGALG00000027035	ENSGALG00000027771	ENSGALG00000025842	ENSGALG00000028537	ENSGALG00000026978	ENSGALG00000026383
ENSGALG00000035252	ENSGALG00000028872	ENSGALG00000027891	ENSGALG00000027963	ENSGALG00000027583	ENSGALG00000027963	ENSGALG00000026300	ENSGALG00000028612	ENSGALG00000027035	ENSGALG00000026490

Continued on next page

Supplementary Table 4 (*continued*) The top 1% highly expressed genes at each skeletal muscle

Extraocular	Longissimus dorsi	Pectoralis thoracica	Quadratus lumbrorum	Quadriceps	Tibialis anterior	Gastrocnemius	Soleus	Extensor digitorum longus	Flexor digitorum brevis
ENSGALG000 00035334	ENSGALG000 00029150	ENSGALG000 00028537	ENSGALG000 00028537	ENSGALG000 00027771	ENSGALG000 00028520	ENSGALG000 00026383	ENSGALG000 00028872	ENSGALG000 00027583	ENSGALG000 00026978
ENSGALG000 00035506	ENSGALG000 00029500	ENSGALG000 00028872	ENSGALG000 00028612	ENSGALG000 00027963	ENSGALG000 00028537	ENSGALG000 00026490	ENSGALG000 00029150	ENSGALG000 00027771	ENSGALG000 00027035
ENSGALG000 00035996	ENSGALG000 00029585	ENSGALG000 00029150	ENSGALG000 00028872	ENSGALG000 00028537	ENSGALG000 00028612	ENSGALG000 00026978	ENSGALG000 00029500	ENSGALG000 00027891	ENSGALG000 00027142
ENSGALG000 00036229	ENSGALG000 00029606	ENSGALG000 00029500	ENSGALG000 00029150	ENSGALG000 00028612	ENSGALG000 00028872	ENSGALG000 00027035	ENSGALG000 00029585	ENSGALG000 00027963	ENSGALG000 00027583
ENSGALG000 00036774	ENSGALG000 00029837	ENSGALG000 00029585	ENSGALG000 00029203	ENSGALG000 00028872	ENSGALG000 00029150	ENSGALG000 00027583	ENSGALG000 00029837	ENSGALG000 00028520	ENSGALG000 00027891
ENSGALG000 00036790	ENSGALG000 00030436	ENSGALG000 00029606	ENSGALG000 00029500	ENSGALG000 00029150	ENSGALG000 00029203	ENSGALG000 00027963	ENSGALG000 00030436	ENSGALG000 00028612	ENSGALG000 00027963
ENSGALG000 00036955	ENSGALG000 00030878	ENSGALG000 00029837	ENSGALG000 00029606	ENSGALG000 00029203	ENSGALG000 00029500	ENSGALG000 00028537	ENSGALG000 00030878	ENSGALG000 00028872	ENSGALG000 00028520
ENSGALG000 00037441	ENSGALG000 00031723	ENSGALG000 00030247	ENSGALG000 00029837	ENSGALG000 00029500	ENSGALG000 00029585	ENSGALG000 00028612	ENSGALG000 00031723	ENSGALG000 00029150	ENSGALG000 00028612
ENSGALG000 00037716	ENSGALG000 00031890	ENSGALG000 00030436	ENSGALG000 00030436	ENSGALG000 00029837	ENSGALG000 00029837	ENSGALG000 00028872	ENSGALG000 00031890	ENSGALG000 00029203	ENSGALG000 00028749
ENSGALG000 00037805	ENSGALG000 00032002	ENSGALG000 00030878	ENSGALG000 00030878	ENSGALG000 00030436	ENSGALG000 00030436	ENSGALG000 00029203	ENSGALG000 00032002	ENSGALG000 00029500	ENSGALG000 00028774
ENSGALG000 00038672	ENSGALG000 00032079	ENSGALG000 00031723	ENSGALG000 00030985	ENSGALG000 00030878	ENSGALG000 00030878	ENSGALG000 00029500	ENSGALG000 00032079	ENSGALG000 00029837	ENSGALG000 00028872
ENSGALG000 00040098	ENSGALG000 00032142	ENSGALG000 00031890	ENSGALG000 00031723	ENSGALG000 00030985	ENSGALG000 00031723	ENSGALG000 00029837	ENSGALG000 00032142	ENSGALG000 00030436	ENSGALG000 00029203
ENSGALG000 00041091	ENSGALG000 00032404	ENSGALG000 00032002	ENSGALG000 00031826	ENSGALG000 00031723	ENSGALG000 00031890	ENSGALG000 00030436	ENSGALG000 00032404	ENSGALG000 00030878	ENSGALG000 00029500

Continued on next page

Supplementary Table 4 (*continued*) The top 1% highly expressed genes at each skeletal muscle

Extraocular	Longissimus dorsi	Pectoralis thoracica	Quadratus lumbrorum	Quadriceps	Tibialis anterior	Gastrocnemius	Soleus	Extensor digitorum longus	Flexor digitorum brevis
ENSGALG00000041541	ENSGALG00000032456	ENSGALG00000032079	ENSGALG00000031890	ENSGALG00000031826	ENSGALG00000032002	ENSGALG00000030878	ENSGALG00000032456	ENSGALG00000031723	ENSGALG00000029837
ENSGALG00000041826	ENSGALG00000032465	ENSGALG00000032142	ENSGALG00000032002	ENSGALG00000031890	ENSGALG00000032079	ENSGALG00000030985	ENSGALG00000032465	ENSGALG00000031826	ENSGALG00000030436
ENSGALG00000042478	ENSGALG00000033212	ENSGALG00000032403	ENSGALG00000032079	ENSGALG00000032002	ENSGALG00000032142	ENSGALG00000031723	ENSGALG00000033212	ENSGALG00000031890	ENSGALG00000030878
ENSGALG00000042750	ENSGALG00000033271	ENSGALG00000032404	ENSGALG00000032142	ENSGALG00000032079	ENSGALG00000032404	ENSGALG00000031826	ENSGALG00000033271	ENSGALG00000032002	ENSGALG00000031723
ENSGALG00000043064	ENSGALG00000033446	ENSGALG00000032456	ENSGALG00000032404	ENSGALG00000032142	ENSGALG00000032456	ENSGALG00000031890	ENSGALG00000033446	ENSGALG00000032079	ENSGALG00000031890
ENSGALG00000043234	ENSGALG00000033700	ENSGALG00000032465	ENSGALG00000032456	ENSGALG00000032404	ENSGALG00000032465	ENSGALG00000032002	ENSGALG00000033884	ENSGALG00000032142	ENSGALG00000032002
ENSGALG00000043379	ENSGALG00000033884	ENSGALG00000033212	ENSGALG00000032465	ENSGALG00000032456	ENSGALG00000033212	ENSGALG00000032079	ENSGALG00000034083	ENSGALG00000032404	ENSGALG00000032079
ENSGALG00000043768	ENSGALG00000034083	ENSGALG00000033271	ENSGALG00000033212	ENSGALG00000032465	ENSGALG00000033271	ENSGALG00000032142	ENSGALG00000035079	ENSGALG00000032456	ENSGALG00000032142
ENSGALG00000045362	ENSGALG00000034615	ENSGALG00000033446	ENSGALG00000033271	ENSGALG00000032595	ENSGALG00000033446	ENSGALG00000032287	ENSGALG00000035252	ENSGALG00000032465	ENSGALG00000032404
ENSGALG00000046937	ENSGALG00000035079	ENSGALG00000033884	ENSGALG00000033446	ENSGALG00000033212	ENSGALG00000033884	ENSGALG00000032456	ENSGALG00000035334	ENSGALG00000033212	ENSGALG00000032456
ENSGALG00000047114	ENSGALG00000035252	ENSGALG00000034083	ENSGALG00000033700	ENSGALG00000033271	ENSGALG00000034083	ENSGALG00000032465	ENSGALG00000035506	ENSGALG00000033271	ENSGALG00000032465
ENSGALG00000047227	ENSGALG00000035334	ENSGALG00000035079	ENSGALG00000034083	ENSGALG00000033446	ENSGALG00000035079	ENSGALG00000033212	ENSGALG00000035584	ENSGALG00000033446	ENSGALG00000034083
ENSGALG00000047395	ENSGALG00000035506	ENSGALG00000035252	ENSGALG00000035079	ENSGALG00000033700	ENSGALG00000035252	ENSGALG00000033446	ENSGALG00000035996	ENSGALG00000034083	ENSGALG00000035079

Continued on next page

Supplementary Table 4 (*continued*) The top 1% highly expressed genes at each skeletal muscle

Extraocular	Longissimus dorsi	Pectoralis thoracica	Quadratus lumbrorum	Quadriceps	Tibialis anterior	Gastrocnemius	Soleus	Extensor digitorum longus	Flexor digitorum brevis
ENSGALG00000047425	ENSGALG00000035584	ENSGALG00000035334	ENSGALG00000035252	ENSGALG00000034083	ENSGALG00000035334	ENSGALG00000033700	ENSGALG00000036229	ENSGALG00000035079	ENSGALG00000035252
ENSGALG00000047506	ENSGALG00000035996	ENSGALG00000035506	ENSGALG00000035334	ENSGALG00000035079	ENSGALG00000035506	ENSGALG00000034083	ENSGALG00000036774	ENSGALG00000035252	ENSGALG00000035334
ENSGALG00000047890	ENSGALG00000036229	ENSGALG00000035996	ENSGALG00000035506	ENSGALG00000035252	ENSGALG00000035584	ENSGALG00000035079	ENSGALG00000036790	ENSGALG00000035334	ENSGALG00000035506
ENSGALG00000048310	ENSGALG00000036774	ENSGALG00000036229	ENSGALG00000035584	ENSGALG00000035334	ENSGALG00000035996	ENSGALG00000035252	ENSGALG00000036955	ENSGALG00000035506	ENSGALG00000035584
ENSGALG00000048383	ENSGALG00000036790	ENSGALG00000036774	ENSGALG00000035996	ENSGALG00000035506	ENSGALG00000036229	ENSGALG00000035334	ENSGALG00000037050	ENSGALG00000035584	ENSGALG00000035996
ENSGALG00000048612	ENSGALG00000036955	ENSGALG00000036790	ENSGALG00000036229	ENSGALG00000035836	ENSGALG00000036774	ENSGALG00000035506	ENSGALG00000037441	ENSGALG00000035996	ENSGALG00000036229
ENSGALG00000048623	ENSGALG00000037441	ENSGALG00000036955	ENSGALG00000036774	ENSGALG00000035996	ENSGALG00000036790	ENSGALG00000035584	ENSGALG00000037716	ENSGALG00000036229	ENSGALG00000036774
ENSGALG00000048682	ENSGALG00000037716	ENSGALG00000037441	ENSGALG00000036790	ENSGALG00000036229	ENSGALG00000036955	ENSGALG00000035836	ENSGALG00000037805	ENSGALG00000036774	ENSGALG00000036790
ENSGALG00000048761	ENSGALG00000037805	ENSGALG00000037716	ENSGALG00000036955	ENSGALG00000036774	ENSGALG00000037050	ENSGALG00000035996	ENSGALG00000037864	ENSGALG00000036790	ENSGALG00000036955
ENSGALG00000049118	ENSGALG00000037864	ENSGALG00000037805	ENSGALG00000037050	ENSGALG00000036790	ENSGALG00000037441	ENSGALG00000036229	ENSGALG00000038672	ENSGALG00000036955	ENSGALG00000037050
ENSGALG00000049267	ENSGALG00000038672	ENSGALG00000038672	ENSGALG00000037441	ENSGALG00000036955	ENSGALG00000037716	ENSGALG00000036774	ENSGALG00000038813	ENSGALG00000037441	ENSGALG00000037441
ENSGALG00000049412	ENSGALG00000038813	ENSGALG00000038813	ENSGALG00000037716	ENSGALG00000037050	ENSGALG00000037805	ENSGALG00000036790	ENSGALG00000040098	ENSGALG00000037716	ENSGALG00000037716
ENSGALG00000049450	ENSGALG00000038884	ENSGALG00000038884	ENSGALG00000037805	ENSGALG00000037441	ENSGALG00000037864	ENSGALG00000036955	ENSGALG00000040969	ENSGALG00000037805	ENSGALG00000037805

Continued on next page

Supplementary Table 4 (*continued*) The top 1% highly expressed genes at each skeletal muscle

Extraocular	Longissimus dorsi	Pectoralis thoracica	Quadratus lumbrorum	Quadriceps	Tibialis anterior	Gastrocnemius	Soleus	Extensor digitorum longus	Flexor digitorum brevis
ENSGALG00000049716	ENSGALG00000040098	ENSGALG00000040098	ENSGALG00000037864	ENSGALG00000037716	ENSGALG00000038672	ENSGALG00000037050	ENSGALG00000040995	ENSGALG00000037864	ENSGALG00000038672
ENSGALG00000049726	ENSGALG00000040969	ENSGALG00000040969	ENSGALG00000038672	ENSGALG00000037805	ENSGALG00000038813	ENSGALG00000037441	ENSGALG00000041091	ENSGALG00000038672	ENSGALG00000038813
ENSGALG00000049865	ENSGALG00000040995	ENSGALG00000040995	ENSGALG00000038813	ENSGALG00000037880	ENSGALG00000040098	ENSGALG00000037716	ENSGALG00000042257	ENSGALG00000038813	ENSGALG00000039216
ENSGALG00000049938	ENSGALG00000041091	ENSGALG00000041091	ENSGALG00000040098	ENSGALG00000038672	ENSGALG00000040969	ENSGALG00000037805	ENSGALG00000042478	ENSGALG00000039977	ENSGALG00000040098
ENSGALG00000050278	ENSGALG00000042257	ENSGALG00000042478	ENSGALG00000040995	ENSGALG00000040098	ENSGALG00000040995	ENSGALG00000037880	ENSGALG00000042750	ENSGALG00000040098	ENSGALG00000041091
ENSGALG00000050376	ENSGALG00000042478	ENSGALG00000042750	ENSGALG00000041091	ENSGALG00000040995	ENSGALG00000041091	ENSGALG00000038672	ENSGALG00000043379	ENSGALG00000040995	ENSGALG00000041555
ENSGALG00000050477	ENSGALG00000042750	ENSGALG00000043379	ENSGALG00000042257	ENSGALG00000041091	ENSGALG00000042257	ENSGALG00000038813	ENSGALG00000043768	ENSGALG00000041091	ENSGALG00000041826
ENSGALG00000050515	ENSGALG00000043379	ENSGALG00000043768	ENSGALG00000042478	ENSGALG00000042257	ENSGALG00000042478	ENSGALG00000040098	ENSGALG00000044125	ENSGALG00000041555	ENSGALG00000042478
ENSGALG00000051082	ENSGALG00000043768	ENSGALG00000044125	ENSGALG00000042750	ENSGALG00000042478	ENSGALG00000042750	ENSGALG00000041091	ENSGALG00000045362	ENSGALG00000042478	ENSGALG00000042750
ENSGALG00000051119	ENSGALG00000044125	ENSGALG00000045362	ENSGALG00000043379	ENSGALG00000042750	ENSGALG00000043379	ENSGALG00000042478	ENSGALG00000046828	ENSGALG00000042750	ENSGALG00000043379
ENSGALG00000051266	ENSGALG00000045362	ENSGALG00000046828	ENSGALG00000043768	ENSGALG00000043379	ENSGALG00000043768	ENSGALG00000042750	ENSGALG00000047359	ENSGALG00000043379	ENSGALG00000043768
ENSGALG00000051509	ENSGALG00000046828	ENSGALG00000047425	ENSGALG00000044125	ENSGALG00000043768	ENSGALG00000045362	ENSGALG00000043379	ENSGALG00000047425	ENSGALG00000043768	ENSGALG00000045362
ENSGALG00000051517	ENSGALG00000047359	ENSGALG00000048064	ENSGALG00000045362	ENSGALG00000045362	ENSGALG00000046828	ENSGALG00000043768	ENSGALG00000048064	ENSGALG00000045362	ENSGALG00000046828

Continued on next page

Supplementary Table 4 (*continued*) The top 1% highly expressed genes at each skeletal muscle

Extraocular	Longissimus dorsi	Pectoralis thoracica	Quadratus lumbrorum	Quadriceps	Tibialis anterior	Gastrocnemius	Soleus	Extensor digitorum longus	Flexor digitorum brevis
ENSGALG00000051617	ENSGALG00000047425	ENSGALG00000048310	ENSGALG00000046828	ENSGALG00000046828	ENSGALG00000047425	ENSGALG00000045362	ENSGALG00000048310	ENSGALG00000046828	ENSGALG00000047425
ENSGALG00000051870	ENSGALG00000048064	ENSGALG00000048546	ENSGALG00000047425	ENSGALG00000047425	ENSGALG00000048064	ENSGALG00000046828	ENSGALG00000048546	ENSGALG00000047425	ENSGALG00000048064
ENSGALG00000051946	ENSGALG00000048310	ENSGALG00000048612	ENSGALG00000048064	ENSGALG00000048064	ENSGALG00000048310	ENSGALG00000047425	ENSGALG00000048612	ENSGALG00000048064	ENSGALG00000048310
ENSGALG00000052142	ENSGALG00000048546	ENSGALG00000048682	ENSGALG00000048310	ENSGALG00000048310	ENSGALG00000048546	ENSGALG00000048064	ENSGALG00000048682	ENSGALG00000048310	ENSGALG00000048612
ENSGALG00000052338	ENSGALG00000048612	ENSGALG00000049412	ENSGALG00000048546	ENSGALG00000048546	ENSGALG00000048612	ENSGALG00000048310	ENSGALG00000049412	ENSGALG00000048612	ENSGALG00000048682
ENSGALG00000052797	ENSGALG00000048682	ENSGALG00000049538	ENSGALG00000048612	ENSGALG00000048612	ENSGALG00000048682	ENSGALG00000048612	ENSGALG00000049865	ENSGALG00000048682	ENSGALG00000049412
ENSGALG00000053046	ENSGALG00000049412	ENSGALG00000049865	ENSGALG00000048682	ENSGALG00000048682	ENSGALG00000049412	ENSGALG00000048682	ENSGALG00000049891	ENSGALG00000049412	ENSGALG00000049450
ENSGALG00000053246	ENSGALG00000049865	ENSGALG00000049891	ENSGALG00000049412	ENSGALG00000049412	ENSGALG00000049865	ENSGALG00000049412	ENSGALG00000051517	ENSGALG00000049865	ENSGALG00000049865
ENSGALG00000053477	ENSGALG00000049891	ENSGALG00000051517	ENSGALG00000049865	ENSGALG00000049865	ENSGALG00000049891	ENSGALG00000049865	ENSGALG00000051870	ENSGALG00000051082	ENSGALG00000051082
ENSGALG00000053705	ENSGALG00000051517	ENSGALG00000051598	ENSGALG00000049891	ENSGALG00000049891	ENSGALG00000051517	ENSGALG00000051152	ENSGALG00000052338	ENSGALG00000051517	ENSGALG00000051517
ENSGALG00000053794	ENSGALG00000051870	ENSGALG00000051870	ENSGALG00000051517	ENSGALG00000051517	ENSGALG00000051870	ENSGALG00000051517	ENSGALG00000052797	ENSGALG00000051598	ENSGALG00000051598
ENSGALG00000053991	ENSGALG00000052338	ENSGALG00000052338	ENSGALG00000051870	ENSGALG00000051870	ENSGALG00000052338	ENSGALG00000051870	ENSGALG00000053046	ENSGALG00000051870	ENSGALG00000051870
ENSGALG00000054033	ENSGALG00000052797	ENSGALG00000052797	ENSGALG00000052338	ENSGALG00000052338	ENSGALG00000052797	ENSGALG00000052338	ENSGALG00000053055	ENSGALG00000052338	ENSGALG00000052338

Continued on next page

Supplementary Table 4 (*continued*) The top 1% highly expressed genes at each skeletal muscle

Extraocular	Longissimus dorsi	Pectoralis thoracica	Quadratus lumbrorum	Quadriceps	Tibialis anterior	Gastrocnemius	Soleus	Extensor digitorum longus	Flexor digitorum brevis
ENSGALG00000054060	ENSGALG00000053046	ENSGALG00000053046	ENSGALG00000052797	ENSGALG00000052797	ENSGALG00000053046	ENSGALG00000052797	ENSGALG00000053625	ENSGALG00000052797	ENSGALG00000052797
ENSGALG00000054189	ENSGALG00000053625	ENSGALG00000053991	ENSGALG00000053046	ENSGALG00000053046	ENSGALG00000053625	ENSGALG00000053046	ENSGALG00000053991	ENSGALG00000053046	ENSGALG00000053046
ENSGALG00000054874	ENSGALG00000053991	ENSGALG00000054033	ENSGALG00000053991	ENSGALG00000053991	ENSGALG00000053991	ENSGALG00000053991	ENSGALG00000054033	ENSGALG00000053991	ENSGALG00000053991
ENSGALG00000054951	ENSGALG00000054033	ENSGALG00000055094	ENSGALG00000054033	ENSGALG00000054033	ENSGALG00000054033	ENSGALG00000054033	ENSGALG00000055094	ENSGALG00000054033	ENSGALG00000054033
